# *Aspergillus flavus* originated pure compound as a potential antibacterial

**DOI:** 10.1186/s12866-021-02371-3

**Published:** 2021-11-20

**Authors:** Saeed Ullah Khattak, Ghosia Lutfullah, Zafar Iqbal, Jamshaid Ahmad, Irshad Ur Rehman, Yanbin Shi, Saima Ikram

**Affiliations:** 1 Center of Biotechnology and Microbiology, University of Peshawar, Peshawar, KPK Pakistan; 2 Department of Agricultural Chemistry, University of Agriculture, Peshawar, KPK Pakistan; 3 School of Pharmacy, Lanzhou University, Lanzhou, 730000 People’s Republic of China

**Keywords:** Bioactive metabolite, Molecular docking, NMR, LCMS, In-silico, *Staphylococcus aureus*, *Proteus vulgaris*

## Abstract

**Problem Background:**

Penicillin was the first and most famous fungal secondary metabolite used as broad spectrum antibiotic that revolutionarised pharmaceutical research and also saved millions of lives. The over optimistic belief in 1967 that sufficient antibiotics had been discovered to defeat infectious diseases was quickly crashed with the appearance of multidrug resistant (MDR) bacteria in 1990s. This has posed a serious threat to mankind. Although scientists are making efforts to synthesize and discover new antibiotics there are not enough new drugs in pharmaceutical pipeline to beat the pace at which MDR bacteria are emerging. In view of this there is an urgent and serious medical need for new bioactive compounds to be discovered to treat infections caused by MDR pathogens. The present study is aimed to investigate the antibacterial potential of *Aspergillus flavus* originated compounds that may act as drug leads to treat future infections.

**Methodology:**

Among the 6 isolated fungal strains from the rhizosphere of *Mentha piperetta*, one was processed for isolation of secondary metabolites on the basis of preliminary antibacterial testing. Observation of morphological and microscopic features helped in identification of the fungal strain as *Aspergillus flavus*. Potato Dextrose Agar (PDA) medium was used for fungal growth while Czapec Yeast Broth (CYB) medium was used for production of fungal metabolites. Column chromatography technique was utilized for purification of compound from crude fungal extract and the mass of the compound was determined using Liquid Chromatography Mass Spectrometry (LCMS) method. Structure elucidation of the pure compound was performed using 500 Varian Nuclear Magnetic Resonance (NMR) machine. Docking was performed using Glide SP algorithm. Agar well diffusion method was used to determine the invitro antibacterial potential of the compound against two MDR bacterial strains i.e. *Staphylococcus aureus* and *Proteus vulgaris*. For this a total of 4 dose concentrations i.e. (100, 250, 500, 1000 μg mL^− 1^) of the compound were prepared and applied to bacterial strains on Mueller Hinton agar using tetracycline as control.

**Results:**

The chemical name of the purified compound from *A. flavus* was determined as (2E)-3-[(3S, 4R)-8-hydroxy-3, 4-dimethyl-1-oxo-3, 4-dihydro-1H-2- benzopyran-7-yl] prop-2-enoic acid with the formula C_14_H_14_O_5_ and exact mass of 262.08. The in-Silico analysis showed that this compound has the potential to inhibit the binding pocket of *S. aureus* TyrRS (1JII) with docking score of − 8.67 Kcal mole^− 1^. The results obtained from invitro experiments were encouraging as at 1000 μg mL^− 1^ the compound showed 58.8% inhibition against *S. aureus* and 28% inhibition against *P. vulgaris*.

**Conclusions:**

The pure compound with formula C_14_H_14_O_5_ and exact mass of 262 exhibited antibacterial potential both insilico and invitro against both Gram negative and Gram positive bacteria. The compound was more active against *S. aureus* in comparison to *P. vulgaris*. From the obtained results it is concluded that this compound can be used as potent antibacterial candidate but further studies will be needed prior to its use as antibiotic.

## Introduction

The emergence of multi-drug resistant pathogens results in serious and life-threatening human infections. This results in pressing demands for the entry of new antibiotics into the existing cohorts [1]. Bioactive compounds with antimicrobial properties may be chemically synthesized; however, nature is the best source of potential bioactive secondary metabolites that can be used as a potential drug leads [2–4]. Fungi, in this regard are the best resource, with more than 1.5 million species among which only 5% have been explored [3, 5]. Numerous compounds of fungal origin have already been reported to demonstrate antibacterial, antifungal, cytotoxic, and phytotoxic properties [6].

With the discovery of penicillin in 1928, the importance of fungal species as a source of antibiotics was realized and the search for new antibiotics, especially of fungal origin, has since gained immense attention. Many structurally complex and therapeutically important secondary metabolites have been recovered from fungi in recent years [7, 8]. In fungal kingdom, *Aspergillus* genus has the highest innovation index regarding the development of biologically active secondary metabolites [9, 10]. These isolated secondary metabolites display a wide range of biological activity including antifungal, antibacterial, and anticancer [11, 12].

Besides species variations, the habitats in which fungi grow also influence the type of bioactive secondary metabolites in these fungal species [13]. For example, Schulz et al. [14] reported that plant associated fungi produces greater proportion of secondary metabolites as compared to soil associated fungi. The available evidence suggests that *Aspergillus* species from plant rhizosphere are excellent producers of antimicrobial metabolites [15]. In this study we focused on the purification and structure elucidation of compounds produced by rhizosphere inhabiting *A. flavus* and to investigate its antibacterial potential. Here we are reporting the insilico and invitro antibacterial potential of compound 262 against MDR strains of *Staphylococcus aureus* and *Proteus vulgaris*.

## Materials and methods

### Isolation of fungal strains

The rhizosphere samples were collected from the soil surrounding roots of *M*e*ntha piperetta*. The plant material and rhizosphere samples were collected from the garden of Centre of Biotechnology and Microbiology, University of Peshawar. Fungal strains were isolated through soil dilution plate method by inoculating on Potato Dextrose Agar (PDA) Acumedia® for ten (10) days at 28 °C. Fungal strains were then sub-cultured and purified.

### Preliminary antibacterial testing of the isolates

Antibacterial potential of the isolated fungal strains was tested against eight pathogenic bacterial stains i.e. Xanthomonas oryzae*, Klebsiella pneumonia, Bacillus subtilus,* Proteus vulgaris*,* Staphylococcus aureus*,* Escherichia coli*,* Salmonella typhi and *Shigella flexeneri*. For culturing of bacterial strains nutrient broth medium (Sigma Aldrich, Germany) was prepared and 10 mL media was transferred to each test tube. The test tubes were plugged with a cotton plug and carefully placed in a beaker. Nutrient agar medium was also prepared in a flask which was also plugged with a cotton plug. Both nutrient agar and nutrient broth media along with petriplates wrapped in paper, metallic cork borer and cotton swabs were transferred to autoclave for sterilization. After autoclaving nutrient agar medium was poured into petriplates and after solidification these petriplates were tightly sealed with parafilm. These petriplates were labeled and were kept in incubator overnight for sterility checking. For culturing of pathogenic bacteria into broth medium a small portion of bacterial culture was transferred with the help of sterile wire loop into the test tubes containing sterile nutrient broth medium and were tightly plugged with a cotton plug. The test tubes were labeled accordingly, were placed in a beaker and transferred to incubator for incubation at 37 °C for 18 h.

After incubation bacterial cultures along with petriplates were transferred to LFH. With the help of sterile cotton swabs a uniform lawn of experimental bacteria were made on nutrient agar plates and allowed for 5 min. With the help of sterile metallic cork borer pustules were taken from the 7 days old fungal cultures and were placed on the lawn of bacteria in the nutrient agar plates. After covering with parafilm the petriplates were transferred to incubator for 24 h at 37 °C for determination of zone of inhibition. This experiment was performed in triplicate to record an accurate zone of inhibition and to identify bioactive fungal strains.

### Identification of bioactive fungal strains

The colony morphology of the pure fungal isolates and pigmentation was observed by growing the fungus on different growth media. Slide culture technique was utilized to study structure of fruiting bodies and arrangement of spores using light microscope (100–200 magnification) after staining the slide with lactophenol blue stain. The fungal strain was further confirmed from department of plant pathology, The University of Agriculture, Peshawar.

### Culturing of fungi for production of metabolites

For metabolites production, the fungal strain was inoculated on Czapec Yeast Broth (CYB) medium, containing 1% Glucose, 1% Peptone, 0.05% KCl, 0.05% MgSO_4_·7H_2_O, and 0.001% FeSO_4_·7H_2_O; pH 7.3 ± 0.2, supplemented with 2% glucose and 3% starch and transferred to shaking incubator for 14 days at 28 °C and 150 rpm.

### Extraction of metabolites from liquid culture medium

After completion of the incubation period 200–250 μl of 40% concentrated HCl was added to each flask for separating the media components and for improving their settlement. Electrical blender was used to grind the mycelia, and then equal volume of ethyl acetate was added to individual flask. This blend was followed by mixing and shaking from time to time for 30 min. Cheese cloth was used to filter mycelia, and organic layer was recovered by using separating funnel followed by washing with 2 M brine solution to remove impurities. Anhydrous sodium sulphate (Na_2_SO_4_) was added to dehydrate organic layer. Finally, the organic layer that contains the required metabolites (in crude form) was re-filtered and shifted to rotary evaporator at 45 °C for concentrating the metabolites. The dried crude extract was then recovered from the flask of rotary evaporator by rinsing it with methanol.

### Isolation of fungal metabolites using column chromatography techniques

To separate the crude metabolites into individual components the fungal crude extract was dissolved in an appropriate solvent (methanol) and was kept for 10 min for complete solubility. Slurry of the sample was prepared in a small amount of silica gel of 60 mesh size using mortar and pestle. The sample was mixed thoroughly till the solvent is evaporated and the sample is completely dried.

The glass column was washed thoroughly with water and later with 75% ethanol and allowed to dry. Silica bed was formed by soaking silica in 100% *n*–Hexane solution. It was then carefully poured into the glass column. After settling of silica at the bottom of column, the solvent was collected in a beaker at the bottom of the column. The sample was then poured with the help of a glass funnel on to the silica bed inside the column. After passing 100% *n*–Hexane the polarity of the solvent system was gradually increased by adding ethyl acetate while thin layer chromatography (TLC) was regularly performed to check for isolated compounds [16]. The separated spots (bands) that appeared on TLC plates were then visualized in UV light at wavelength of 254 and 366 nm (nm).

### LC–MS/HPLC analysis

Liquid chromatography mass spectrometry (LCMS) (Waters 2795HT HPLC system) was used for further purification and investigation of fungal compound. The instrument is equipped with Waters 2998 photodiode array detector joined to a Micro–Mass ZQ spectrometer for the detection of UV between 200 and 400 nm. The solvent system for both analytical and preparative runs comprised of HPLC grade ultrapure water, HPLC grade methanol (MeOH) and HPLC grade Acetonitrile (CH_3_CN). To the solvent system, formic acid (0.055%) was also added. For analytic run, total volume of 20 μl was injected initially followed by gradual increase for purification.

### Nuclear magnetic resonance (NMR) spectroscopy

1D (^1^H, ^13^C) and 2D (HSQC, HMBC, COSY) NMR spectra were obtained using 500 MHz Varian NMR machine. Chemical shifts in ppm were referenced to the internal tetra methyl cylane (TMS). The unity of 500 MHz stands 500 for ^1^H and 125 for ^13^C NMR. 6 mg of pure compound (262) was mixed in 0.65 mL of deuterated chloroform and a series of one dimensional and two dimension NMR analyses were performed on 500 MHz NMR Spectrometer.

### In-Silico analysis

#### Protein preparation

Crystal structure of *S. aureus* TyrRS (PDB code: 1JII) were taken from protein databank. Structure was prepared using protein preparation wizard of Maestro to add hydrogen atoms, fixed side chains and loops, and to generate disulphide bonds. The protonation states of amino acids were then identified at physiological pH 7.4 using PROPKA. Force field OPLS-2005 was used for protein optimization [17–19].

### LigPrep

Compound 262 was extracted from fungal origin. The structure was characterized by NMR and 2D structure was then generated. Compound 262 was prepared using LigPrep module of the Maestro. 20 tautomers were generated. Epik was used to protonate the ligand at pH 7.4. Force field OPLS-2005 was used for ligand preparation [19, 20].

### Molecular docking

For docking studies, Glide SP and Glide-IFD algorithm was used in Maestro with flexible ligand docking [21, 22]. Receptor Grid box was generated around crucial residues in a catalytic pocket. OPLS-2005 was used for generating different confirmations of the ligand [17]. Twenty different poses were generated at the end of the docking.

### In vitro antibacterial activity of pure compound

To investigate the antibacterial activity of the pure compound two pathogenic bacterial strains i.e. *P. vulgaris* and *S. aureus* were used. The bacterial strains under study was previously isolated, identified and maintained in the lab. Agar well diffusion method was used for determination of antibacterial activity of the pure compound. For preparing fresh cultures, the bacterial strains were inoculated in 1 mL sterile Mueller- Hinton broth (MHB) medium autoclaved at 121 °C. After inoculation, these pathogenic bacterial strains were kept at 37 °C for 24 h. Mueller- Hinton agar (MHA) medium was prepared according to the manufacturer’s instructions; autoclaved and 22 mL of MHA medium was dispensed in each Petri plate. After solidification of the agar medium, wells of desired number were made in each plate using sterile metal borer maintaining appropriate distance. The turbidity of the bacterial strains was adjusted using 0.5 McFarland’s turbidity standard and 20 μL of each experimental organism was inoculated on nutrient agar plates to prepare a uniform lawn. Using sterile di-methyl sulfoxide (DMSO) a stock solution of 10 mg mL^− 1^ was prepared which was further used to prepare dose concentrations of 100, 200, 250, 500 and 1000 μg mL^− 1^. After preparation, 80 μL volume of each concentration was added in the respective well with the help of a micropipette. Tetracycline with a dose concentration of 250 μg mL^− 1^ was used as reference antibiotic. For allowing proper diffusion of the pure compound into the nutrient agar medium, the plates were kept at 25 °C for some time in laminar flow hood so that compound could be absorbed into the medium and then shifted to incubator at 37 °C for 24 h for measuring the inhibition zone.

## Results

### Identification of fungal strains

For identification of fungal strain, different traits were taken into account e.g., structure of hyphae, colony structure and arrangement of spores. Furthermore, the fungal strain was also grown on PDA and Czapec Dox Agar (CDA) medium to observe the morphology of fungal colony. The isolate was then sent to the Department of Plant Pathology, University of Agriculture Peshawar for further confirmation and identified as *A. flavus*.

### Result of preliminary antibacterial testing of *A. flavus*

In comparison to other isolated fungal strains, *A. flavus* was found more bioactive against the test bacterial stains. The highest zone of inhibition in millimeters was recorded against *Staphylococcus aureus* (32.5*)* followed by *Proteus vulgaris* (29). A zone of 27, 24, 22.5, 20, 15 and 9 was recorded for *Shigella flexeneri, Escherichia coli, Bacillus subtilis, Klebsiella pneumoniae, Salmonella typhi* and *Xanthomonas oryzae* respectively.

### Purification and mass determination of the compound

**Fig. 1:**
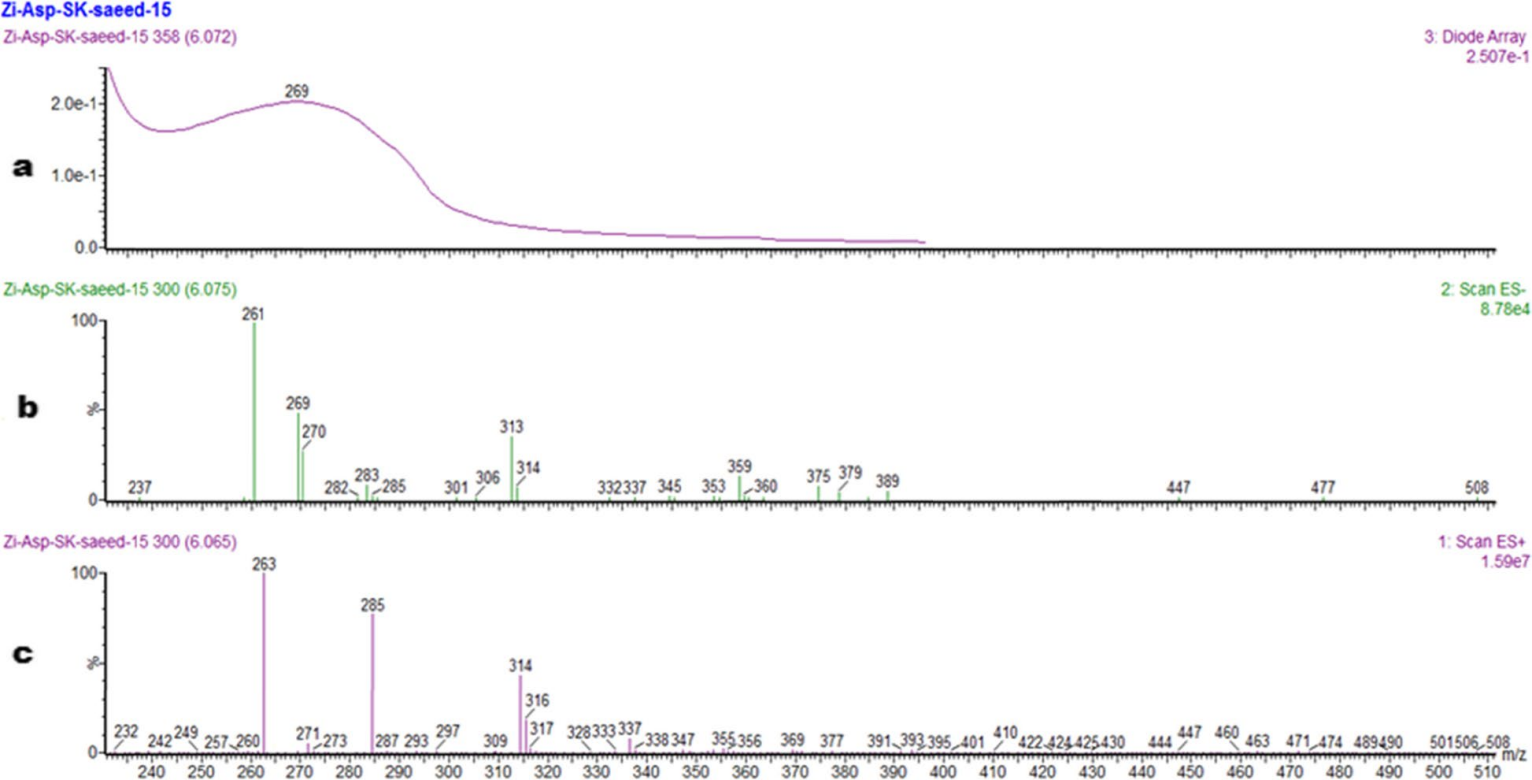
LCMS chromatogram of the compound showing UV profile (**a**), ES^−^ profile (**b**) and ES+ profile (**c**)

Solvent system comprising of Ethyl acetate-*n*-Hexane was used for purification of fungal compounds starting from 5:95 v/v of Ethylacetate:*n*-Hexane using flash column chromatography. Polarity of the solvent was gradually increased and corresponding fractions were obtained. At a polarity of 85:15 v/v of Ethyl acetate:*n*-Hexane (4 L), 10 mg of compound was purified. Water’s LCMS-HPLC system was used for further purification and analysis of the compound. The LCMS chromatogram (Fig. 1) of the compound showed that the compound eluted at Rt 6.06 min with UV profile of 269 (λ_max_) and ES^−^ and ES^+^ profile of 261 and 263 [M]H^+^ respectively, calculated for the compound with the formula C_14_H_14_O_5_ and exact mass of 262.08.

### LCMS chromatogram of compound (262)

#### Description of compound (262)

The HR–ESI–MS and ^13^C NMR (Broad Band and DEPT) were used to establish the molecular formula of the known compound. HR–ESI–MS showed pseudo molecular ion peaks [M^+^] at *m/z* 262.1112 [M]H^+^ and 284.2121 [M]Na^+^ that correspond to molecular formula C_14_H_14_O_5_ [Calculated as C_14_H_14_O_5_ + Na = 284.2121]. The spectrum got from IR spectroscopy showed occurrence of hydroxyl group (3350 cm^− 1^), carbonyl group at (1740 cm^− 1^), and aromatic ring (1635 and 1381 cm^− 1^). The absorption maxima in the UV spectrum (320 nm) also showed the occurrence of an aromatic ring. The pure compound (262) at a concentration of 6 mg was mixed in 0.65 mL of deuterated chloroform and a series of 1-dimensional and 2-dimensional NMR analyses were performed on 500 MHz NMR Spectrometer.

The Proton NMR represented total of eight proton signals. Two signals at *δ* 1.36 and *δ* 1.39 corresponded to two CH_3_ with an integration of three protons. The remaining signals represented the integration of single proton The two aromatic protons were signaled at *δ* 7.21 and *δ* 7.52 as doublets The two signals at chemical shift 6.5 ppm and 6.9 ppm (both doublets) revealed as alkenyl hydrogens attached to the carboxyl group.

**Fig. 2:**
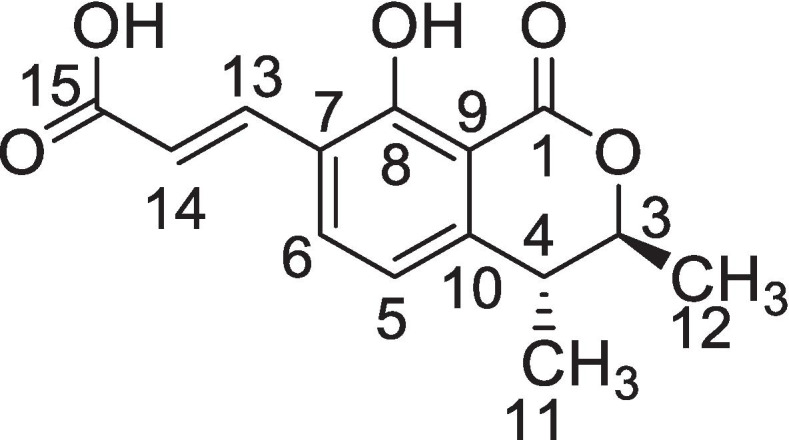
Structure of the compound 262

The 1D- ^13^C NMR gave 14 signals that corresponded to 14 carbons skeleton of the compound, exhibiting 4 quaternary carbons at *δ* 125.5, *δ* 161.3,*δ* 125.5 and *δ* 138.1while 2 carbonyl carbons at *δ* 170.2 and 168.9 respectively. The 2D-HSQC exhibited 8 protonated carbons as given in Table 1**.** The two dimensional (^1^H–^1^H COSY and HMBC) represented different correlations and the structure is elucidated as given in Fig. 2. The NMR data of known compound 262 is given in Table 1. In ^1^H–^1^H COSY, the methine proton of H–3 showed correlation with the proton of C–4, also with the methyl protons of C–12, while the H–4 showed correlation with the methyl protons of C–11. The proton H–5 showed correlation the proton of C–6, and the proton H–13 showed correlation the proton of C–14 and vice versa. While in HMBC correlations were compared with the compound already reported in literature. The 2D (^1^H–^1^H COSY and HMBC) alone with UV and IR were compared with the values in literature [23].

**Table 1:** Chemical Shifts of Known Compound (262). Data Obtained from 500 MHz Varian NMR

Carbon No.	Multiplicity (DEPT)	^**13**^C–NMR	^**1**^H (***J*** = Hz)
1	C=O	170.2	–
2	–	–	–
3	CH	83.1	4.56 m
4	CH	38.8	3.87 m
5	CH	120	7.21d (*J* = 10.0 Hz)
6	CH	137.2	7.52d (*J* = 10.0 Hz)
7	–C–	125.5	–
8	–C–	161.2	–
9	–C–	125.5	–
10	–C–	138.1	–
11	CH_3_	17.4	1.12d(*J* = 10.0 Hz)
12	CH_3_	19.7	1.23d(*J* = 10.0 Hz)
13	CH	140	6.98d(*J* = 10.0 Hz)
14	CH	122	6.5.23d(*J* = 10.0 Hz)
15	C=O	168.9	–

### Molecular docking simulation

Two different docking algorithms including Glide-SP and Glide-IFD in Maestro were used with flexible and rigid ligand docking for compound 262. Active site residues of 1JII (PDB ID) were observed as shown in the crystal structure. The most favorable conformations based on its occupancy in the active site and its interactions with binding pocket residues and docking score were then analyzed (Table 2).

**Table 2:** Docking score of the pure compound 262 with *S. aureus* tRNA synthetase

Molecule	Glide SP kcal/mol	Glide IFD kcal/mol
262 (molecular name)	−6.35	−8.67

**Fig. 3:**
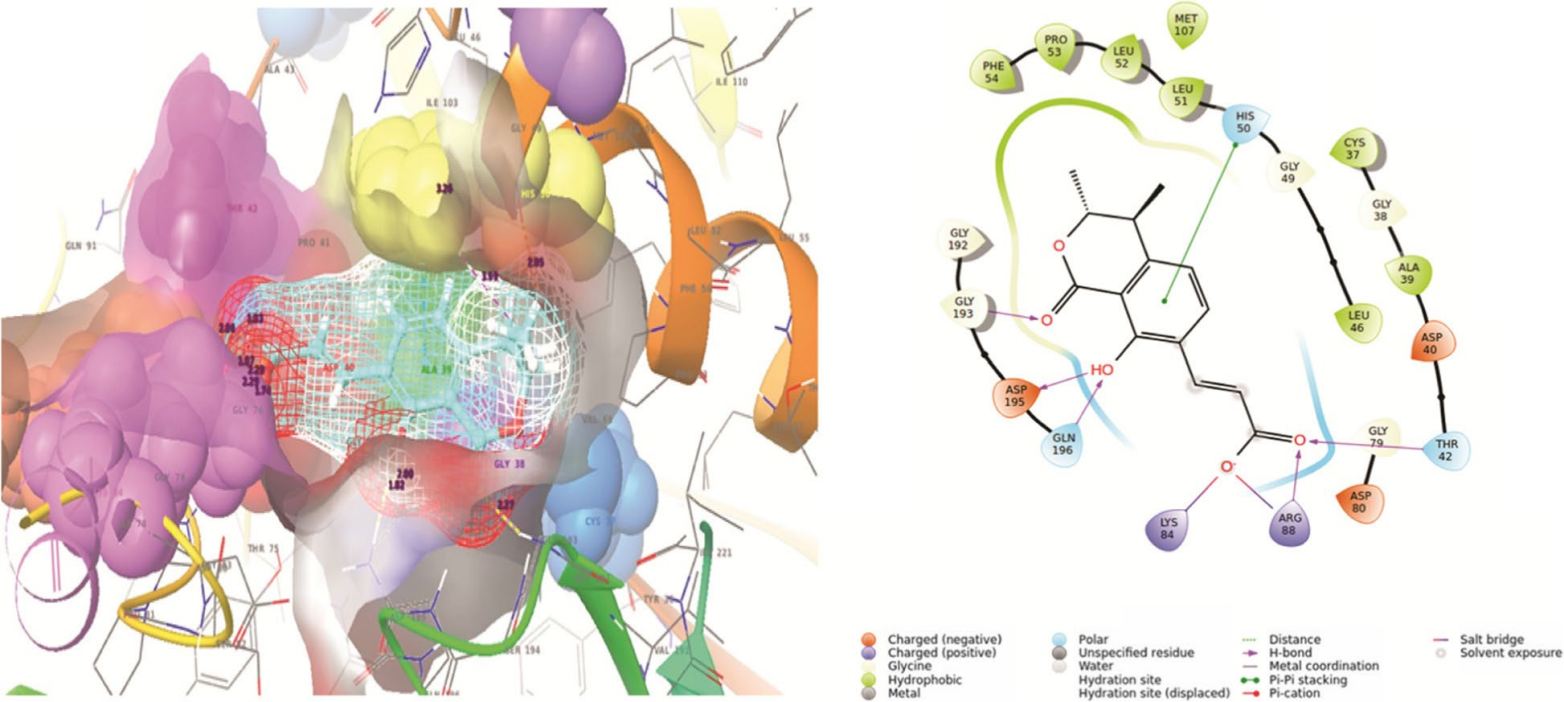
Docking of compound 262 in the active site of tRNA synthetase showing interaction with key amino acids residues along-with bond lengths

In Glide SP Compound 262 showed interactions with amino acids residues including Thr-42, His-50, Leu-51, Le-52, Pro-53, Phe-54, Gln-196, Asp-195, Gly-193, Gly-192, Lys-84, Arg-88, Asp-80. Leu-51, 52, Pro-53, Phe-54, Gly-192 and Gly-193, showed non covalent interactions. Gly-193, Asp-195, Gln-196, Arg-88, Thr-42 showed hydrogen bonding with compound 262, while His-50 showed Pi-Pi interactions. Furthermore, Lys-84 and Arg-88 are also involved in the formation of salt bridges (Fig. 3).

**Fig. 4:**
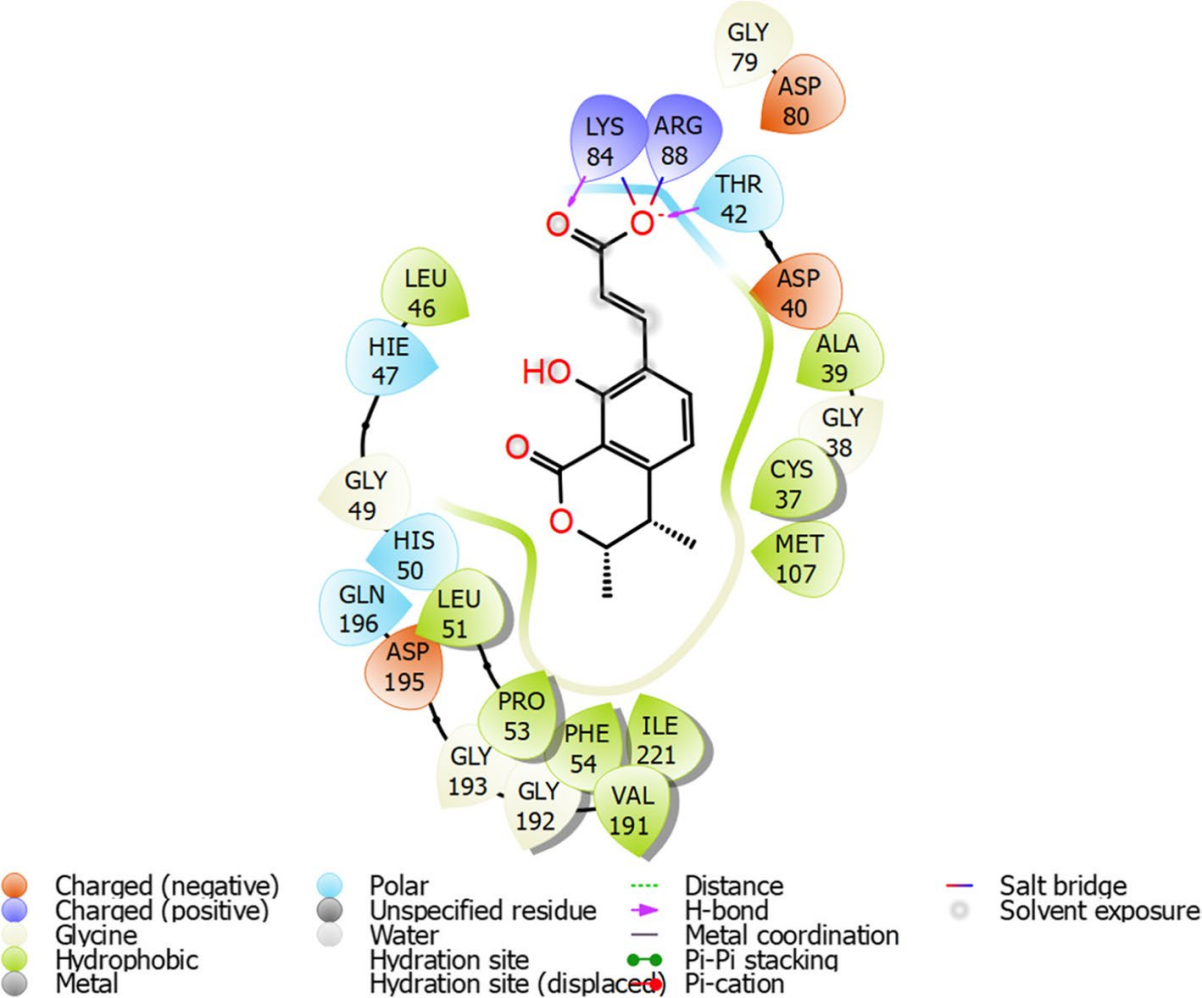
Interaction map of compound 262 with amino acids residues of the active site pocket of *S. aureus* tyrosyl-tRNA synthetase. Figure legend is shown below the map

In Glide IFD docking score of compound increase with the dock score of − 8.67 kcal/mol. Lys84 and Arg88 maintain salt bridges and hydrogen bond in IFD. Almost similar type of interactions were observed in IFD as we observed in Glide SP Fig. 4.

### In vitro antibacterial potential of the pure compound

**Fig. 5:**
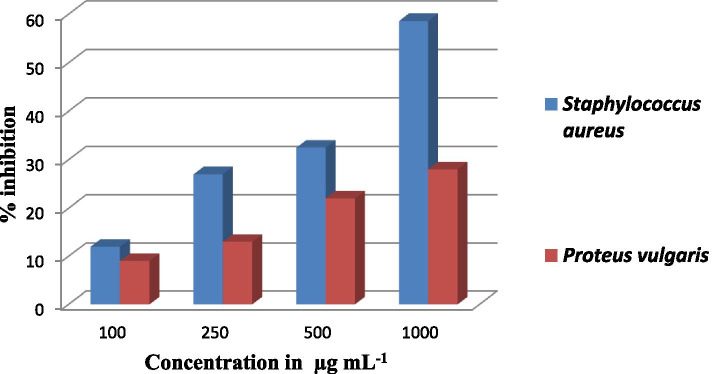
Antibacterial activity of pure compound against *S. aureus* and *P. vulgaris*

Agar well diffusion method was used to investigate the antibacterial potential of the purified compound against two MDR pathogenic bacterial strains i.e. *S. aureus* and *P. vulgaris*. Using sterile di-methyl sulfoxide (DMSO) a stock solution of 10 mg mL^− 1^ was prepared which was further used to prepare dose concentrations of 100, 200, 250, 500 and 1000 μg mL^− 1^. At 100 μg mL^− 1^12% inhibition was recorded against *S. aureus* while 9% inhibition was recorded against *P. vulgaris*. 27 and 13% inhibition was noted at 250 μg mL^− 1^ of the compound against *S. aureus* and *P. vulgaris* respectively. At 500 and 1000 μg mL^− 1^ of the compound 32.5 and 58.6% inhibition was recorded against *S. aureus* while 22 and 28% inhibition was recorded against *P. vulgaris* (Fig. 5)*.*

## Discussion

Growth and chemical diversity of fungi is greatly affected by the nature of habitat because to adjust according to particular environment they produce different metabolites. In the current study rhizosphere inhabiting *A. flavus* was processed for isolation of secondary metabolites with antibacterial properties. Within rhizosphere, a number of biochemical and physiological interactions takes place between plant roots, soil fauna and other microbial communities [24]. Available data suggests that in comparison to soil born microbial species, fungal strains from plant rhizosphere provides a better chance for isolation of bioactive metabolites with antimicrobial properties [10, 15, 24, 25]. The present study deals with samples from soil, rhizosphere and different plant parts. Further, they were processed for isolation of fungal strains. *A. flavus* isolated from rhizosphere of mint plant exhibited promising antimicrobial activity. The strain was subjected to purification of pure compound. From the list of different isolated compounds, we are reporting the one identified as as (2E)-3-[(3S, 4R)-8-hydroxy-3, 4-dimethyl-1-oxo-3, 4-dihydro-1H-2- benzopyran-7-yl] prop-2-enoic acid. The structure of the compound and the values when searched online in dictionary of natural products showed similar mass and UV with that of a polyketide derivative reported from endophytic fungus *Pestalotiopsis* species [23]. The identified compound discussed here was reported earlier from *Pestalotiopsis* species. The current study is reporting this compound isolated from *A. flavus.* The compound was subjected to in-silico and in-vitro antibacterial activity and the results revealed promising results. Crystal structure of *S. aureus* TyrRS was downloaded from PDB to analyse the *in-silico* behaviour of 2E)-3-[(3S, 4R)-8-hydroxy-3, 4-dimethyl-1-oxo-3, 4-dihydro-1H-2- benzopyran-7-yl] prop-2-enoic acid. Our results showed strong interaction with key amino acids residues of target proteins with a dock score of − 8.67 Kcal mole^− 1^ (Fig. 5).

For in-vitro screening of the compound, two MDR bacterial pathogens i.e. *S. aureus* and *P. vulgaris* were selected. The compound showed activity against both the bacterial strains by inhibiting the growth of *S. aureus* and *P. vulgaris* by 58.6 and 28% respectively. These results are in accordance with the previously published data as reported by Alfaki et al. (2019) which states that compounds isolated from terrestrial *Aspergillus* species have the potential to inhibit MDR gram positive and gram negative bacterial strains [26]. In the previous years different compounds from the species of genus *Aspergillus* have been reported to have broad spectrum antibacterial activity among which some are currently used as commercial antibiotics [27–29]. Among different compounds isolated from *Aspergillus* species a compound called CJ-17,665 showed activity against different MDR bacteria including multi-drug resistant *S. aureus* (MDRSA).

The purified compound and its inhibitory activity against MDR bacterial strains presented here projects the possibility of this compound as a new antibacterial drug. The compound has the ability to retard the growth of both gram positive and gram negative bacterial strains by showing a prominent zone of inhibition. The purified compound may be considered as potential antibacterial candidate to be used against the two test MDR bacterial strains. However, further studies are needed to confirm our findings and a number of selective toxicity assays needs to be perform before its application as a new antibacterial compound.

### Study limitations

This study has a few limitations. Primarily the amount of the purified compound was only 10 mg so as a result the sample size is very small. The compound was tested against only one gram positive and one gram negative bacterial strains. Secondly, due to limited amount of the compound minimum inhibitory concentrations (MICs) could not be performed. Selective toxicity of the compound was not tested. In future the study will be expanded by optimizing the culture conditions that will help to purify greater amount to the compound. Sample size will be increased by increasing the number of test bacterial strains. Selective toxicity of the compound will be tested using animal models which will not only help to enhance the impact but will also help in further refinement of this study.

## Conclusions

The present study concludes from results of insilico and invitro experiments that the compound purified from *A. flavus* has the potential to inhibit the growth of two test bacterial strains. In comparison to *P. vulgaris* the compound more actively inhibited the growth of *S. aureus*. Further, it is concluded that this compound can be used as potent antibacterial candidate but further studies will be needed prior to its use as antibiotic.

## Data Availability

Lab related data and other analysis sheets can be requested from the corresponding author.

## Abbreviations

*A. flavus*: Aspergillus flavus
*P. vulgaris*: *Proteus vulgaris*
*S. aureus*: *Staphylococcus aureus*
PDA: Potato Dextrose Agar
CYB: Czapec Yeast Broth
LCMS: Liquid Chromatography Mass Spectrometry
NMR: Nuclear Magnetic Resonance
MDR: Multidrug resistant
TLC: Thin layer chromatography
UV: Ultraviolet
HPLC: High performance liquid chromatography
MeOH: Methanol
CH_3_CN: Acetonitrile
TyrRS: Tyrosyl-tRNA synthetase
